# Improving the Sustainability of Processing By-Products: Extraction and Recent Biological Activities of Collagen Peptides

**DOI:** 10.3390/foods12101965

**Published:** 2023-05-12

**Authors:** Shumin Xu, Yuping Zhao, Wenshan Song, Chengpeng Zhang, Qiuting Wang, Ruimin Li, Yanyan Shen, Shunmin Gong, Mingbo Li, Leilei Sun

**Affiliations:** 1 College of Life Science, Yantai University, No. 30, Qing Quan Road, Yantai 264005, China; lmm199791@163.com (S.X.); water15689@163.com (Y.Z.); cpzhang1997@163.com (C.Z.); wqt1981979471@163.com (Q.W.); 17861135192@163.com (R.L.); yyshenqx1999@163.com (Y.S.); gong1394@foxmail.com (S.G.); sllshd1991@163.com (M.L.); 2 Marine Biomedical Research Institute of Qingdao, No. 23, Hong Kong East Road, Qingdao 266073, China; songwsh@ouc.edu.cn

**Keywords:** by-products of aquatic animals, collagen peptides, bioactivity, industrial application

## Abstract

Society and consumers are increasingly concerned about food safety and the sustainability of food production systems. A significant amount of by-products and discards are generated during the processing of aquatic animals, which still needs to be fully utilized by the food industry. The management and sustainable use of these resources are essential to avoiding environmental pollution and resource waste. These by-products are rich in biologically active proteins, which can be converted into peptides by enzymatic hydrolysis or fermentation treatment. Therefore, exploring the extraction of collagen peptides from these by-products using an enzymatic hydrolysis technology has attracted a wide range of attention from numerous researchers. Collagen peptides have been found to possess multiple biological activities, including antioxidant, anticancer, antitumor, hypotensive, hypoglycemic, and anti-inflammatory properties. These properties can enhance the physiological functions of organisms and make collagen peptides useful as ingredients in food, pharmaceuticals, or cosmetics. This paper reviews the general methods for extracting collagen peptides from various processing by-products of aquatic animals, including fish skin, scales, bones, and offal. It also summarizes the functional activities of collagen peptides as well as their applications.

## 1. Introduction

**Figure 1:**
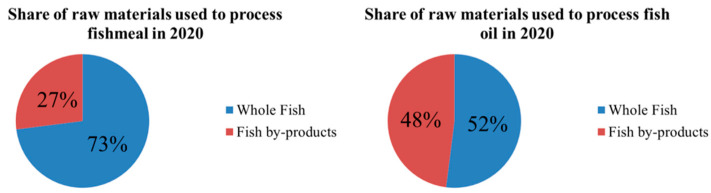
Share of raw materials used to process fishmeal and fish oil in 2020.

The demand for fish and its products grows as the world population increases and human consumption behavior changes [1]. These processing by-products also put a lot of pressure on the environment if they are discarded and buried. However, these by-products are rich in protein, mainly collagen, and can be well processed into valuable products. The Food and Agriculture Organization of the United Nations (FAO) has recently published the 2022 edition of The State of World Fisheries and Aquaculture. According to the report, the total production of fisheries and aquaculture reached a new peak of 214 million tons in 2020, with 178 million tons of aquatic animals being produced. Fish by-products make up around 70% of processed fish products and typically comprise of fish heads (9–12% of total fish weight), offal (12–18%), skin (1–3%), bones (9–15%), and scales (approximately 5%). The Marine Raw Materials Organization reports that some fish by-products can be utilized to produce fish oil and fishmeal (Figure 1), but others are discarded as waste, resulting in economic losses and environmental pollution. However, these by-products are rich in protein, primarily collagen, and can be well processed into valuable products.

Collagen is a major extracellular protein, accounting for about 30% of total protein, and is mainly found in various fibrous tissues such as skin, bone, blood vessels, tendons, muscles, cornea, and ligaments [2,3]. To date, 28 types of collagens have been identified, with type I collagen being the most abundant animal protein (90%) [4]. A repetitive amino acid sequence at the molecular level characterizes collagen. The sequence consists mainly of (Gly-X-Y)_n_, where glycine (Gly) makes up about 1/3 of the collagen peptide chain, X,Y are arbitrary amino acids other than glycine. Typically, the classical composition is proline (Pro) at the X-position and 4-hydroxyproline (Hyp) at the Y-position [3,5]. Collagen has unique physical properties, including homogeneity, tensile strength, flexibility, various bioactive functions, and controlled enzymatic properties [6]. Collagen plays an important role in various biological processes, such as promoting cell proliferation, differentiation, and migration [7]. It also regulates and mediates coagulation while facilitating wound tissue repair and regeneration [8,9]. Degradation of collagen can lead to various diseases and syndromes, such as osteoarthritis and joint pain, hair abnormalities, skin laxity, and muscle weakness.

Many studies have found that collagen has a certain nutritional value. However, realizing its efficacy and value is difficult because of its considerable molecular weight and strong bonding, which cannot be quickly digested, absorbed, and utilized by humans or animals. The collagen peptide is the product of enzymatic digestion of collagen or gelatin and has a noticeable improvement in digestion and absorption, nutrition, and functional effects compared with collagen [10,11]. The market for collagen peptides has seen significant growth both domestically and internationally. The bioactive functions of collagen peptides are increasingly widely recognized, including antioxidative [12,13,14,15], anti-fatigue [16,17,18], hypoglycemic activity [19,20], hypolipidemic activity [21,22], antimicrobial attributes [23,24], and other activities [25,26]. The collagen peptides market is expected to grow at a Compound Annual Growth Rate (CAGR) of 5.42% to exceed USD 22,622 million by 2027 [27]. The utilization of collagen peptides is rising as dietary supplements in food and beverages and as bioactive ingredients in cosmetics [28,29], nutraceuticals [30], and pharmaceuticals [31,32].

The objectives of this review are (1) to examine current approaches on the preparation of collagen peptides from food processing by-products, focusing on their advantages and disadvantages, (2) to summarize the biological activity of collagen peptides, and (3) to discuss the applications and future challenges related to collagen peptides in the food and cosmetic industries.

## 2. Processing Approaches for the Generation of Collagen Peptides

**Figure 2:**
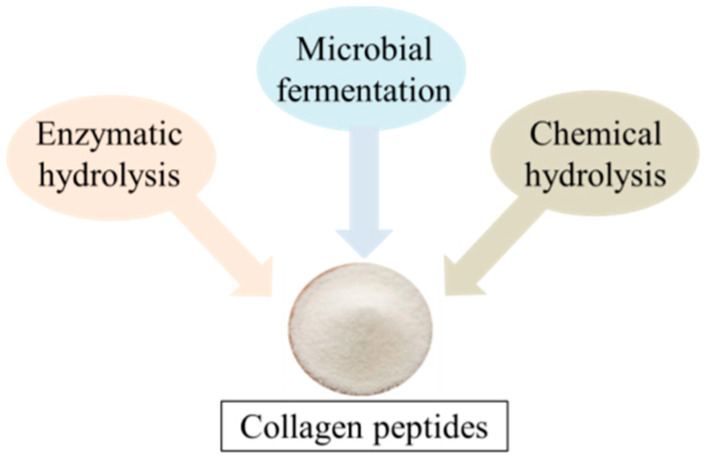
Diagram of the different methods used for the release of collagen peptides.

**Figure 3:**
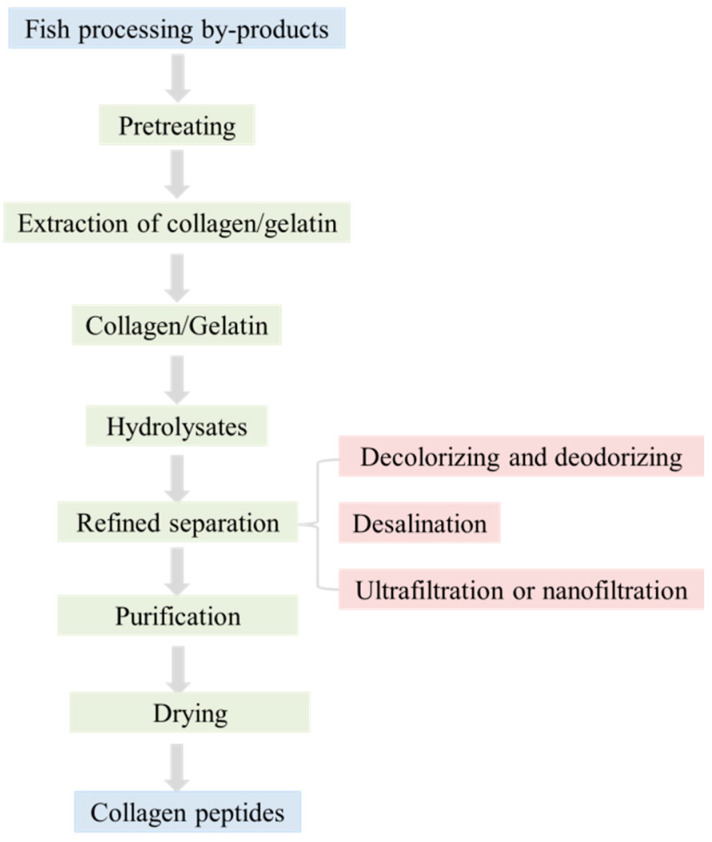
Schematic diagram for preparation of collagen peptides.

There are three primary ways to prepare collagen peptides (Figure 2). The first method involves obtaining bioactive peptides with various functions by enzymatically digesting and hydrolyzing collagen (Figure 3) [15]. The second method requires extracellular enzymes produced by microbial fermentation to prepare collagen peptides [23,33]. Finally, the third chemical hydrolysis method is to prepare collagen peptide by acid-base catalytic decomposition of peptide bond, which is a method to improve the functional properties of protein.

In the long chain of amino acids of proteins, peptide fragments are not biologically active. Only after enzymatic digestion can many biologically active peptides be released and function. Proteolysis is the most commonly used method for the preparation of bioactive peptides. Enzymatic digestion refers to using specific enzymes to carry out the corresponding chemical reaction to eliminate some particular structures. Enzymes are commonly classified as either exonucleases or endonucleases. Exonucleases, such as flavourzyme [34], aminopeptidase, and carboxypeptidase, target the peptide bonds at the end of the peptide chain. On the other hand, endonucleases like alkaline proteases [14,15,35], trypsin [31], and papain [36,37], act on specific regions within the peptide chain. Different proteases have different cleavage sites and degrees of hydrolysis (Table 1) [19]. More studies have been reported on the preparation of collagen peptides using ultrasound [37,38,39] assisted enzymatic digestion, resulting in improved yields and activity. It was found that microwave assisted or steam jet treatment under appropriate conditions could improve the yield and activity of bovine bone collagen peptides. Zhang et al. [40] used steam explosion to treat bovine bone and found that the protein recovery was improved, and the molecular weight of the collagen peptides was significantly reduced. The extracted collagen peptides still had a good calcium binding capacity (44.7 µg/mg) and osteoblast proliferative activity (126.7%) without changing the amino acid composition. Perhaps this method could also be applied to fish bones, fish heads, and other by-products to improve protein recovery.

Microbial fermentation is a process that uses a variety of enzymes synthesized and secreted during the growth of microorganisms to degrade proteins and produce specific small-molecule active peptides [41]. The degree of enzymatic digestion depends on the protein source, strain, fermentation temperature, fermentation time, and pH [42]. Compared to enzymatic hydrolysis, some enzyme-producing strains may be toxic to the organism, making purifying microbial fermentation products more difficult. Although this method is cost-effective and productive for protein preparation, it is yet to be widely used in large-scale production due to technical limitations [41].

Acid extraction mainly uses low ionic concentrations of acidic conditions to impregnate the raw material, thus destroying the intermolecular salt bonds and Schiff bases while causing fiber swelling and dissolution. The primary solvents used are hydrochloric or sulfurous acid, phosphoric acid, sulfuric acid, acetic acid, citric acid, and formic acid [43]. The standard treatment agents used for alkali collagen extraction are lime, sodium hydroxide, sodium carbonate, etc. Since the alkali extraction is easy to cause peptide bond hydrolysis, the molecular weight of the obtained hydrolysis product is relatively low. However, the violent reaction makes it easy to cause environmental pollution and destroy the product’s nutrients. Although the acid-base method is simple, the reaction environment is more extreme, which is not conducive to maintaining biological activity. The acidic or alkaline waste solution produced after extraction must be treated appropriately. Otherwise, some harmful by-products will be made and will cause environmental pollution [43,44].

As can be seen from Table 2, the most commonly used method for the preparation of collagen peptides is enzymatic hydrolysis, including single enzyme hydrolysis and multiple enzyme complex hydrolysis.

In addition, fish-derived collagen peptides often suffer from a heavy fishy taste. The methods of deodorization can be classified as sensory masking, physical, chemical, biological, or a combination of several methods [60]. Han Na Cho et al. [61] used solid-phase microextraction (SPME) and solvent-assisted flavor evaporation (SAFE) combined with gas chromatography-mass spectrometry (GC-MS-O) to identify odour compounds from marine collagen peptides and gelatin, exploring effective removal methods. The current study showed that *Lactobacillus plantarum* fermentation was the most effective in removing the odour from the collagen peptides.

## 3. Biological Characteristics of Collagen Peptide

Collagen peptides are a class of small molecule amino acid sequences with various biological functions. Hydrolyzing the protein with proteinases disrupts its primary structure, even though the active peptide does not exhibit biological activity in the intact protein structure. The organism benefits from the release of the free peptide for its health. Bioactive peptides have superior absorption mechanisms and functional properties that cannot be compared to amino acids [62]. Collagen peptides have been found to exhibit various biological activities, such as hypotensive and blood sugar-regulating effects, antioxidant properties, anti-inflammatory and immunomodulatory actions, anti-thrombotic activity, calcium absorption promotion, antibacterial effects, and skin-condition improving properties [56].

### 3.1. Anti-Atherosclerotic Activity

Atherosclerosis, a lipid-driven chronic inflammatory disease that forms plaques in medium and large arteries, is a significant cause of ischemic heart disease and stroke. It occurs mainly in the bends or branches of these arteries, leading to the blockage or rupture of the vessels. As such, it poses one of the greatest threats to human health through cardiovascular disease [63]. A recent study published in a sub-publication of The Lancet assessed the prevalence of carotid atherosclerosis and risk factors in 21 national and regional populations worldwide: nearly 2 billion people will have carotid atherosclerosis in 2021. Atherosclerosis has seriously threatened the human quality of life, health, and longevity [64]. Therefore, effective treatment of atherosclerotic plaques is imperative to slow the disease and save patients’ lives.

More information is needed on the anti-atherosclerosis collagen peptides from a by-product of fish processing. It was found that collagen peptides extracted from the skin of Atlantic salmon inhibited lipid plaque formation in the aortic arch and intimal thickening in the thoracic aorta, showing excellent anti-atherosclerotic effects. Although a mixture of collagen peptides extracted from salmon skin, with a molecular weight ranging from 2 to 30 KDa and composed mainly of glutamine (37%) and myostatin (39%), did not significantly improve carotid atherosclerosis or cardiac structure in hypertensive patients during the short-term intervention, it was found to impact systolic cardiac function positively [35].

Therefore, anti-atherosclerosis is one area of research exploration through collagen peptides prepared from aquatic animals processing by-products. It is challenging to find other peptides that reduce the risk of atherosclerosis through bioinformatics.

### 3.2. Anticoagulant Activity

Excessive blood clotting, known as pathological thrombosis, can lead to serious health issues such as pulmonary infarction, cerebral thrombosis, retinal arteriovenous obstruction, myocardial infarction, and embolisms of the extremities. These conditions have become a leading cause of morbidity and mortality in China. While blood clotting is a necessary physiological defense process of the body, it must be regulated to prevent these harmful outcomes [65]. Surveys show that in recent years, 1–3 out of every 1000 people suffer from thrombosis-like diseases, which seriously endanger human health. Commonly used anticoagulant substances in clinical practice, such as heparin, oral coumarin, streptokinase, and urokinase, can cause side effects like thrombocytopenia and subcutaneous purpuric bleeding [66]. Peptides and some small molecule peptides have been studied for their anticoagulant activity, such as Hirulog-1 [67], Argatroban [68], etc., but few peptides have been studied for their anticoagulant activity in fish collagen peptides.

Anticoagulant activity has been found in oligopeptides isolated from the skin of silver carp [52,69], Yellowfin sole (*Limanda aspera*) frame [70], and mackerel skin [71]. All isolated peptides were tested in vitro and showed the ability to prolong activated partial thromboplastin in a dose-dependent manner. The three oligopeptides of silver carp skin, which are not inactivated by gastrointestinal digestion and are absorbed by Caco-2, inhibited adenosine diphosphate and thrombin-induced platelet aggregation in a dose-dependent manner, however had no effect on platelet release. Although weaker than Argatroban, it also significantly prolonged APTT, TT and PT. In addition, the 60 mg/mL tripeptide showed significantly higher (*p* < 0.05) platelet aggregation inhibitory activity (~30%), similar to the synthetic antithrombotic compounds like aspirin and indomethacin [69].

Peptides with an anticoagulant activity have molecular weights below 3.5 KDa, and most are below 2.5 KDa. Therefore, it seems useful to obtain low molecular weight peptides with a good anticoagulant activity by ultrafiltration, microfiltration, or nanofiltration [72]. Although the anticoagulant activity of collagen peptides, a by-product of fish processing, has not been studied much, those peptides that have been isolated have demonstrated satisfactory results. Therefore, further studies are necessary to find more anticoagulant peptides from fish and to assess whether they show the same side effects as warfarin. In conclusion, based on adequate studies, collagen peptides could be a good natural source of alternatives to anticoagulant drugs.

### 3.3. Antidiabetic Activity

Diabetes mellitus (DM) is a severe chronic metabolic disease characterized by persistent elevation of collective blood glucose levels due to defective insulin secretion and resistance [20]. DM is mainly marked by high blood glucose, with common symptoms such as excessive drinking, polyuria, polyphagia, and lethargy. Long-term persistent high blood glucose will lead to chronic complications, including heart, brain, liver, kidney, spleen, retina, nervous system, skeletal muscle and blood vessels, and other tissues [73]. The dramatic increase in the incidence of diabetes has made it an important global issue. Studies have shown that DM is characterized by long duration, difficulty to cure, and high disability rate, and most patients require long-term drug therapy. There are a wide variety of drugs for the clinical treatment of diabetes, including insulin promoters, insulin sensitizers, biguanides, alpha-glucosidase inhibitors, etc. Clinical diabetes medication has various types, but they may have issues with their effectiveness, how they work, and their adverse side effects. Therefore, searching for new, safe, natural hypoglycemic agents to replace synthetic hypoglycemic agents has become a growing need [74]. The most described antidiabetic peptides are derived from milk and soy protein; however, several recent studies have shown that collagen peptides can be effective in alleviating syndromes associated with diabetes [75]. For example, Zhang et al. [76] reported that a protein hydrolysate (TCP) isolated from the skin of Nile tilapia (*Oreochromis niloticus*) improved the symptoms of “excessive drinking and eating” in diabetic mice, and that a high dose of TCP produced better results than metformin. Similarly, Harnedy et al. [77] reported that protein hydrolysates isolated from Atlantic salmon (*Salmo salar*) skin gelatin and excipients could exhibit in vitro anti-diabetic activity by inducing the release of glucagon-like peptide 1 (GLP-1). Some of the targets of diabetes therapy are glucosidase and alpha-amylase, and therefore hypoglycemic activity is expressed in vitro as the inhibition of α-glucosidase or α-amylase. Most of the antidiabetic peptides have molecular weights below 1 KDa. Collagen peptides produced by collagenase hydrolysis, as assessed by the inhibition of α-amylase, showed antidiabetic activity of 80.45% for CP-5 compared to 75.81% and 71.17% for CP-25 and CP-50, respectively, at a concentration of 1 mg/mL [78]. In a double-blind randomized trial evaluating the effectiveness of collagen peptide (CPT) as a nutritional supplement in patients with T2DM, it was shown that taking 5 g of CPT over the three-month study period significantly reduced fasting blood glucose and glycated hemoglobin in the subjects [79]. The above studies clearly reveal the potential application of fish collagen peptides in the treatment of diabetes. Nevertheless, more clinical trials are still needed to use collagen peptides more rationally in future biomedicine.

### 3.4. Anti-Inflammatory Activity

Inflammation is an immune stress response, a complex and highly regulated defense response of the immune system to pathogenic microbial infections, physical or chemical stimuli, etc. It manifests primarily as redness, swelling, heat, and pain, killing pathogens, limiting illness, and repairing the damage [72]. However, the targeted destruction and assisted repair stage needs to be corrected. In that case, inflammation can cause continuous tissue damage by leukocytes, lymphocytes, and collagen, which can be harmful to the organism and, in severe cases, life-threatening. Anti-inflammatory drugs including steroids, non-steroidal anti-inflammatory drugs, and monoclonal antibodies can be used to combat the inflammatory response [80]. However, many drug treatments are not sufficient to control chronic inflammatory responses and long-term treatment may lead to adverse events [81]. Therefore, the development of natural products with anti-inflammatory properties is essential as for alternative and safe dietary supplements.

A number of collagen peptides derived from fish have now been shown to have anti-inflammatory activity. Most of the anti-inflammatory peptides are derived from algae and molluscs, but they have also been identified in hydrolysates from tuna cooking juice by-products [82], Salmo salar skin [81], and unicorn leatherjacket (*Aluterus monoceros*) [78]. The results showed that Salmo salar skin collagen hydrolysate had anti-inflammatory effects on LPS-induced RAW264.7 cells, among which the QA peptide which showed strong anti-inflammatory activity with an IC_50_ value of 849.3 μM against the LPS-induced NO secretion in macrophages. Similarly, Kumar et al. [78] conducted in vitro anti-inflammatory assays on human red blood cell (HRBC) membranes and found a slight protective effect on HPBC with increasing collagen peptide concentrations, with a maximum membrane protection of 4.01%, 3.66% and 3.27% for CP-5, CP-25, and CP-50 expression at 0.2 mg/mL concentrations, respectively.

### 3.5. Anti-Cancer Activity

With increasing human life expectancy, lifestyle changes, and environmental pollution, cancer has surpassed cardiovascular disease as the second leading cause of human death in incidence and mortality [83]. The American Association for Cancer Research (AACR) recently released the 2022 Progress Against Cancer report at the end of July this year. The report shows that by 2040, the total number of cancer patients worldwide will reach about 28 million, and about 16.2 million patients will die. The FDA approved eight new anti-cancer therapies and expanded the approval of 10 existing ones to treat additional types of cancers between August 2021 and July 2022 [84]. However, the side effects of the drug can cause damage to several areas, including the gastrointestinal tract, skin, and nervous system. Dietary interventions have a unique advantage over anti-cancer drugs in treating cancer or alleviating the suffering caused by cancer because of their low side effects and low cost. Therefore, the development of functional foods with an anti-cancer activity is imperative. Developing healthy food with anti-allergic effects is socially necessary to compensate for the shortage of traditional drug therapy.

In the last few years collagen peptides have also been shown to exhibit anti-cancer activity, with several in vitro studies showing potential in different cancer types [85]. Peptides isolated from the skin of Unicorn leatherjacket fish (*Aluterus monoceros*) showed inhibitory effects on COLO320 cells in vitro [78]. Baehaki et al. [86] hydrolyzed Milk Fish (*Chanos chanos*) skins by collagenase extracted from Bacillus licheniformis F11.4 w and showed the highest activity against HeLa cells at 60 min and the greatest activity against HCT-166 cells at 30 min. The by-product of Barred mackerel was hydrolyzed with actinidin followed by alcalase to produce a 3~10 KDa peptide. At a concentration of 1 mg/mL, the antioxidant and anti-cancer activities were 67.75% and 96.93%, respectively [87]. Yaghoubzadeh et al. [88] separated bioactive peptides by membrane ultrafiltration using alcalase (HA) and flavourzyme (HF) and hydrolyzing rainbow trout skin. The results showed that collagen peptides with a molecular weight less than 3 KDa had the highest inhibitory concentration. The above studies clearly demonstrate that collagen peptides exhibit significant anti-cancer activity in in vitro assays. However, more in-depth studies are needed to investigate the relationship between the chemical structure of the peptides and their anti-cancer activity through reliable in vivo studies to elucidate the health benefits of collagen peptides.

### 3.6. Antihypertensive Activity

Hypertension refers to a clinical syndrome characterized by increased arterial blood pressure (systolic and diastolic) in the body circulation (systolic blood pressure ≥140 mm Hg and diastolic blood pressure ≥90 mm Hg), which may increase the incidence and severity of cardiovascular disease, peripheral arterial disease, heart failure, and stroke with impaired kidney function [89,90]. More than one sixth of the blood pressure-lowering drugs used worldwide are angiotensin I-converting enzyme (ACE) inhibitors, such as captopril, because ACE is the key enzyme in the process of raising blood pressure in humans [91]. Drugs used to control high blood pressure can cause side effects; for this reason, much research has focused on finding alternatives to control or prevent this disease through diet [92]. There is an urgent need for new ACE inhibitors to treat hypertension, and inhibitors from natural sources are promising medicinal and edible supplements due to their efficacy and safety. In the last few years, several researchers have been involved in the development of collagen peptides as ACE inhibitors. Lee et al. [93] found that collagen peptides prepared by trypsin hydrolysis of Chum salmon (*Oncorhynchus keta*) skin exhibited the strongest ACE inhibitory activity. The IC_50_ for ACE inhibitory activity was 18.7 μM for the small molecule heptapeptide Gly-Leu-Pro-Leu-Asn-Leu-Pro with a molecular weight of 770 Da. In vivo experiments demonstrated that Gly-Leu-Pro was comparable to captopril in terms of its antihypertensive effect within 6 h of administration. Since the 1990s, several ACE-inhibiting peptides have been studied in different natural sources such as bovine whey, mushrooms, and walnuts, and most of the anti-hypertensive peptides were found to have molecular weights below 1.5 KDa [72]. Thus, fish processing by-products appear to have great potential as a source of antihypertensive peptides, and notably, collagen peptides deserve higher attention due to their ACE inhibition activity.

### 3.7. Antimicrobial Activity

Excessive use of antibiotics has created a problem by increasing the prevalence of drug-resistant bacteria, a significant public health concern. To address this issue, researchers are exploring biological antimicrobial agents that have broad activity and can be used in food or supplements due to the strict regulations on chemical preservatives [94]. As collagen peptides exhibit hydrophobicity, they can bind to negatively charged surfaces due to electrostatic interactions, penetrate and disrupt membrane structures, leading to the death of bacteria, fungi, parasites, and viruses [95]. Unlike the single-target bactericidal principle of conventional antibiotics, the antimicrobial properties of the peptides are demonstrated with the ability to destroy pathogens by disrupting multiple targets, which can significantly reduce the emergence of resistant bacteria. Their resistance of a wide range of bacteria makes them one of the best alternatives to combination antibiotics [96,97].

Several studies have shown that enzymatic hydrolysis of fish processing by-products can produce peptides with antimicrobial activity. For instance, antibacterial peptide fractions isolated from Atlantic mackerel (*Scomber scombrus*) by-products were reported to inhibit the growth of Gram-positive and Gram-negative bacteria [98]. Maryam Atef et al. [24] also demonstrated that collagen hydrolysates produced by neutrase and papain showed the most effective inhibitory activity against Gram-negative bacteria (*Salmonella strains*), providing enhanced potential for products against food-borne pathogens. Wald et al. [99] used pepsin to prepare antimicrobial hydrolysates from rainbow trout by-products and found that the degree of hydrolysis (DH%) had a considerable effect on the antimicrobial activity, with minimum inhibitory concentrations of 2 mg/mL and 5 mg/mL at 30% DH against the fish farming bacteria *Flavobacterium psychrophilum* and *Renibacterium salmoninarum*. The antimicrobial peptides mainly prolong the lag period of bacterial growth. Furthermore, the wide range of effects suggests that collagen peptides prepared from hydrolyzed fish processing by-products are highly applicable as preservatives in food processing or in fish farming to protect fish health.

### 3.8. Antioxidant Activity

Oxidative stress occurs when there is an imbalance between the production of reactive oxygen species (ROS) and the natural antioxidant capacity of cells. This disruption can lead to abnormal cellular and molecular function caused by a combination of ROS and reactive nitrogen radicals. Excessive production of reactive oxygen species (ROS) in the body can be caused by aging, radiation, alcohol, or drugs. This leads to intracellular proteins, lipids, and DNA damage and triggers apoptosis [100]. Over time, this may result in degenerative diseases associated with aging, like diabetes, cancer, cataractogeneses, Parkinson’s and Alzheimer’s disease. In addition, ROS-induced lipid peroxidation is one of the significant causes of lipid degradation in food and cosmetic matrices [101,102]. The scavenging of ROS is a crucial way to lower health risks associated with oxidation products (such as MDA, carbonylated proteins, etc.). Antioxidants are used to inhibit these free radical-producing compounds. Various antioxidants, such as thiols or vitamin C, vitamin E, uric acid, and lipoic acid, can be used to terminate these oxidation reactions. Butylated hydroxytoluene (BHT), butylated hydroxyanisole (BHA), tert-butylhydroquinone (TBHQ), and propyl hemilactate (PG) are the most commonly used synthetic antioxidants in the food and pharmaceutical industries. Although these synthetic antioxidants have shown greater antioxidant activity than natural antioxidants such as alpha-tocopherol and ascorbic acid, their use has begun to be limited due to the DNA damage and toxicity induced by these compounds [103]. Therefore, searching for antioxidants that significantly inhibit oxidative reactions has become an increasing research focus.

Natural antioxidant peptides are being extracted from different animal or plant species. Several marine species such as mussels, fish, and algae are sources of antioxidant peptides produced by enzymatic hydrolysis [104]. Collagen peptides with antioxidant activity have been developed from a variety of fish processing by-products, such as grass carp scales [14], squid (*Dosidicus gigas*) by-products [105], salmon scales [106], hammerhead shark skin [107], redlip croaker (*Pseudosciaena polyactis*) scales [50], skipjack tuna (*Katsuwonus pelamis*) [108], and lamuru (*Caranx ignobilis*) fishbone [109]. The two most common assays for antioxidant activity of peptides are the scavenging of DPPH radicals and the scavenging of ABTS radicals [72]. These studies found that collagen peptides have better DPPH and ABTS free radical scavenging ability. These studies also found a strong correlation between the amino acid composition and the molecular weight of the peptides and the level of antioxidant activity. In comparison, Wang et al. [50] found that collagen hydrolysate (RSCH) from red fish scales prepared using neutral protease showed the highest hydrolysis (21.36 ± 1.18%) and 2,2-diphenyl-1-picrylhydrazyl (DPPH·) radical scavenging activity (30.97 ± 1.56%) among the six hydrolysis products. Subsequently, the amino acid sequences GPEGPMGLE, EGPFGPEG, and GFIGPTE were identified as the most active against DPPH radicals (EC_50_ 0.59, 0.37 and 0.45 mg/mL), hydroxyl radicals (EC_50_ 0.45, 0.33 and 0.32 mg/mL) and superoxide anion radicals (EC_50_ 0.62, 0.47 and 0.74 mg/mL) showed the strongest scavenging activity.

### 3.9. Skin Improvement

As one of the principal organs of the body and the main barrier, the skin plays a vital role in protecting tissue structures, regulating temperature, and protecting the body from external stimuli such as ultraviolet (UV) light, heat, toxins, and infections [11,110]. Over time, the skin undergoes a natural aging process, from becoming dry and lacking in elasticity, to developing many wrinkles. The mechanism of skin aging is relatively complex, divided into external factors such as UV radiation and pollution and internal factors such as cell metabolism and hormones that lead to the skin’s loss of elastin and collagen [11,111]. Therefore, the use of molecules that can act on these characteristics may be an effective way to improve skin conditions. With the world’s population aging and increasing concerns about skin health, there is a growing interest in active substances that can improve skin. Collagen peptides have attracted much attention due to their excellent biocompatibility, easy digestibility, and transdermal absorption properties. Studies have shown that collagen peptides extracted from fish skin and fish bone effectively improve skin wrinkles and fine lines and brighten skin tone. Much literature confirms the collagen peptides’ physiological activity for anti-aging and skin photoaging prevention. Clinical trials have shown an overall significant increase in skin elasticity in subjects taking the test product. Histological analysis of skin biopsies showed positive changes in skin structure in the test product group, with reduced daylight elasticity gain and improved collagen fibrous tissue [30]. In his study, Wang et al. [112] found that CHs were prepared from Nile tilapia skin by simulating gastrointestinal digestion, and that visual appearance, tissue structure, and matrix homeostasis were significantly improved in mice skin by feeding them to 9-month-old mice for 180 days. In addition, oxidative stress was reduced by increasing the activity of antioxidant enzymes, which was important for the improvement of the skin.

Collagen hydrolysates/peptides derived from fish processing by-products appear to be an efficient and promising functional ingredient of food and cosmetics for the prevention of skin ageing, as it may promote skin improvement by promoting the proliferation of fibroblasts, which are the main cells in the proliferation phase, and the secretion of growth factors to increase skin collagen levels. The use of these discarded fish parts to obtain collagen hydrolysates may contribute to a sustainable circular economy and improve the skin health of consumers. Aquatic animal processing by-products, especially fish skin, have great potential as an essential source of skin-improving active substances and should be widely applied.

### 3.10. Attenuated Obesity

According to the World Health Organization (WHO), which published the European Regional Obesity Report 2022, overweight and obesity have reached “epidemic” proportions in Europe, affecting nearly 59% of adults and one-third of children in Europe [113]. Being overweight and obese poses health risks beyond personal appearance, including disability and premature death. They are the fourth most common risk factors in Europe after hypertension, diet-related risks, and non-communicable diseases like those caused by the smoking of tobacco. These conditions can lead to type II diabetes, heart disease, musculoskeletal complications, and at least 13 types of cancer, according to recent estimates [34]. The pathogenesis of obesity and metabolic disorders is related to the intestinal microbiota, a complex and dynamic ecosystem whose composition affects nutrient acquisition, energy stabilization, and fat storage [34,114]. Studies have shown that *Bifidobacterium* spp. and *Lactobacillus gasseri* can effectively reduce body fat, improving lipid status and glucose homeostasis [115]. Collagen peptides have also been found to have a beneficial effect on obesity. Chotphruethipong et al. [116] found that the hydrolysis of collagen in the skin of defatted bass (*Lates calcarifer*) inhibited the activity of obesity-related enzymes, such as α-amylase and α-glucosidase. Effective anti-obesity effects of the hydrolysate CP, obtained by enzymatic hydrolysis using walleye cod skin in mice fed a high-fat diet, modulates the overall composition of the intestinal microbiota [34]. Currently, anti-obesity drugs are limited by their high cost and the numerous adverse effects associated with their administration. These results suggest that collagen peptides could be further developed as potential adjuvant therapeutic agents for obesity and related metabolic diseases.

Currently, researchers are primarily focused on preparing collagen peptides with high purity and specific peptides with specific bioactivity through isolation and purification processes. However, these methods are not practical for industrial applications due to their high cost. Additionally, there is a lack of research on the interactions between collagen peptides and other nutrients present in food.

## 4. Industrial Application

The share of collagen peptides in the international market has increased rapidly in recent years. It has formed several application areas, including beauty, joint and bone health, sports health, and topical products. The unique functions of collagen peptides, such as promoting skin [11] and bone repair [117], antioxidants [102], and others, determine their diverse applications.

It is also applied in cosmetics to improve skin moisture and elasticity through transdermal absorption, etc. Collagen peptides have gained attention for their antioxidant activity and skin-improving properties. They can effectively scavenge reactive oxygen radicals, which slows down the aging process of the skin [118,119]. As a result, they are commonly used in cosmetic formulations such as lotions, creams, and masks.

Collagen peptides can be used as nutritional supplements or food fortification to regulate human body functions through oral intake [120]. Kumar et al. [121] mixed MCP (0–10%) into biscuit powder and found that the addition of MCP significantly reduced the water holding capacity (*p* < 0.05) but had no significant effect on the oil absorption capacity. In addition, the protein content and antioxidant capacity of the biscuits were significantly increased (*p* < 0.05), and the calorie content was slightly decreased (*p* < 0.05). Therefore, the function of collagen peptides can be used as a nutritional health food option by combining them into other appropriate food systems.

However, the application of collagen peptides, some of which are only in vitro and cell studies, with no clinical trials as a guide, is still a field with unknowns and its protective mechanism has not been fully elucidated. At the same time, due to the influence of molecular weight, amino acids, and its order on the activity of bioactive peptides, collagen peptides still face great challenges in large-scale screening, purification, and production applications. With the deepening of the biological activity of collagen peptides and the gradual maturity of production technology, its safety should also be better guaranteed.

## 5. Conclusions

As discussed in this paper, collagen peptides of aquatic by-products obtained through enzymatic techniques have been shown to have high biological activity and functional properties, which have been widely used in pharmaceuticals, healthcare products, foodstuffs, daily-use chemicals, and other fields. However, there are still problems, such as low levels of high-value transformation, single development, immature scale, and intelligent production technology. Some new extraction processes (ultrasonic, microwave, supercritical extraction, high voltage pulsed electric field technology, etc.) can be applied to improve the extraction rate and industrialization of collagen peptides in combination with each other. In addition, combinations of two or more enzymes during enzymatic digestion have been shown to produce low molecular weight peptides that readily penetrate the intestinal tract, resulting in beneficial health properties. Future research should also focus on exploring protein-rich by-products that are currently underutilized and neglected, as well as their contaminants.

By-products of aquatic animals have high research value in the field of natural bioactive molecules. Fish collagen peptides have been shown to have biological activities such as antioxidant, antibacterial, antitumor, immunomodulatory, and hypoglycemic, and they are widely used in medicinal applications. Although collagen peptides have efficacy in preventing and treating various diseases, further research and clinical trials are still needed to improve the utilization value.

## Figures and Tables

**Table 1:** The active position of some common proteases.

Protease	Cutting Site
Chymotrypsin	Tyr-, Trp-, Phe-, Leu-
Alcalase	Hydrolysis of the carboxyl terminal peptide bond of aromatic amino acid residues or hydrophobic amino acid residues
Pepsin	Hydrolysis of Phe residue, Leu residue carboxyl terminal peptide bond
Trypsin	Hydrolyzing the carboxyl terminal peptide bond of Arg residue and Lys residue
Neutral protease	Hydrolysis of the C-terminal peptide bonds of aromatic amino acid residues such as Tyr, Try and Phe
Flavor protease	Mainly exonucleases that catalyse the hydrolysis of hydrophobic amino acids at the ends of brain chains
Papain	Broad hydrolysis of peptide bonds with preference for hydrophobic side chain amino acids

**Table 2:** Source, preparation methods, and biological activity of collagen peptides.

Source	Preparation Method	Biological Activity	References
Tuna skin	Enzymatic hydrolysis (Alcalase, Neutrase and Savinase)	Antioxidant activity	[45]
Asian sea bass skin	Enzymatic hydrolysis (Papain)	Wound-healing and antioxidant activities	[46]
Sturgeon fish skin	Enzymatic hydrolysis (Trypsin, Alcalase, Neutrase, Flavourzyme, Pepsin, Papain)	Antimicrobial activity	[24]
Swim bladders of giant croaker	Enzymatic hydrolysis (Neutral protease)	Antioxidant activity	[47]
Redlip croaker scales	Enzymatic hydrolysis (Neutral protease)	Antioxidant activity	[48]
Walleye pollock skin	Enzymatic hydrolysis (Flavourzyme and Alcalase)	Attenuated obesity and modulated gut microbiota	[34]
Yellowfin tuna skin	Enzymatic hydrolysis (Alcalase)	Antioxidant activity	[49]
Silver carp skin	Enzymatic hydrolysis (Alcalase)	Antiplatelet activity	[50]
Silver carp skin	Enzymatic hydrolysis (Alkaline protease, Trypsin, Neutral protease and a mixture of alkaline proteaseand trypsin)	Anti-photoaging activity	[51]
Asian bullfrog skin	Ultrasound-assisted enzymatic Hydrolysis (papain)	Antioxidant activity	[37]
Mixed by-products from various fish species	Enzymatic hydrolysis (Alcalasa)	Antioxidant and antimicrobial activities	[52]
Squid skin	Enzymatic hydrolysis (Papain, Alkaline protease, Neutral protease, Acid protease and Compound protease)	Cryoprotective effect	[53]
Skipjack tuna skins	Enzymatic hydrolysis (Trypsin, Neutrase, Papain, Pepsin, and Alcalase)	Antioxidant activity	[54]
Tilapia skin	Enzymatic hydrolysis (Alcalase, Protamex, Flavourzyme, Neutrase, Papain, Bromelain, and Trypsin)	DPP-IV inhibitory activity	[55]
Tilapia skin	Enzymatic hydrolysis (Alcalase)	ACE-inhibitory activity	[56]
Fresh water fish heads	Microbial fermentation (*E. faecium* NCIM5335, *P. acidilactici* FD3, *P*. *acidilactici* NCIM5368)	Antioxidant activity	[57]
Salmon by-products with skin	Bacterial extracellular protease	Antioxidant and anti-freezing activities	[33]
Bigeye tuna skin	Subcritical water hydrolysis	Antioxidant activity	[58]
Bigeye tuna skin	Catalyst-assisted subcritical water hydrolysis	Antioxidant and antimicrobial activities	[59]

## Data Availability

No new data were created or analyzed in this study. Data sharing is not applicable to this article.

## Abbreviations

FAO	Food and Agriculture Organization of the United Nations
CAGR	Compound Annual Growth Rate
USD	United States Dollar
APTT	Activated partial thromboplastin time
TT	Thrombin time
PT	Prothrombin time
DM	Diabetes mellitus
GLP-1	Glucagon-like peptide 1
T2DM	Diabetes mellitus type 2
LPS	Lipopolysaccharide
HRBC	Human red blood cell
AACR	American Association for Cancer Research
FDA	Food and Drug Administration
ACE	Angiotensin Converting Enzyme
IC_50_	Half-maximal inhibitory concentration
DH	Degree of hydrolysis
ROS	Reactive oxygen species
BHT	Butylated hydroxytoluene
BHA	Butylated hydroxyanisole
TBHQ	Tert-butylhydroquinone
PG	Propyl gallate
DPPH	1,1-Diphenyl-2-picrylhydrazyl
ABTS	2,2′-amino-di(2-ethyl-benzothiazoline sulphonic acid-6)ammonium salt
UV	Ultraviolet
WHO	World Health Organization
